# Can regulatory T cells improve outcomes of sensitised patients after HLA-Ab incompatible renal transplantation: study protocol for the Phase IIa GAMECHANgER-1 trial

**DOI:** 10.1186/s12882-023-03157-7

**Published:** 2023-04-28

**Authors:** C Dudreuilh, P Jarvis, N Beadle, I Pilecka, O Shaw, L Gardner, C Scottà, N Mamode, DS Game, A Sanchez-Fueyo, G Lombardi, A Learoyd, A Douiri, A Dorling

**Affiliations:** 1 Centre for Nephrology, Urology and Transplantation, Department of Inflammation Biology, School of Immunology and Microbial Sciences, King’s College London & NIHR Biomedical Research Centre-Transplant Theme, Guy’s Hospital, Great Maze Pond, London, SE1 9RT UK; 2 Centre for Nephrology, Urology and Transplantation, School of Immunology and Microbial Sciences, King’s College London, London, UK; 3 Clinical Trials Unit, King’s College London, London, UK; 4 Guy’s and St Thomas’s Hospital Trust, London, UK; 5 Peter Gorer Department of Immunobiology, King’s College London, London, UK; 6 Department of Transplantation, Guys and St, Thomas’s Hospital NHS Trust, London, UK; 7 Institute of Liver Studies, King’s College London University and King’s College Hospital, London, UK; 8 School of Population Health and Environmental Sciences, King’s College London, London, UK

**Keywords:** Prospective study, Phase IIa, Regulatory T cells, Highly sensitised, Transplantation, Memory immune response, HLA, GMP, Polyclonal expansion

## Abstract

**Background:**

Kidney transplantation is the gold-standard treatment for patients with kidney failure. However, one-third of patients awaiting a kidney transplant are highly sensitized to human leukocyte antigens (HLA), resulting in an increased waiting time for a suitable kidney, more acute and chronic rejection, and a shorter graft survival compared to non-highly sensitised patients. Current standard immunosuppression protocols do not adequately suppress memory responses, and so alternative strategies are needed. Autologous polyclonally expanded regulatory T cells (Tregs) have been demonstrated to be safe in transplant settings and could be a potential alternative to modulate memory immune alloresponses.

**Methods:**

The aim of this trial is to determine whether adoptive transfer of autologous Tregs into HLA sensitised patients can suppress memory T and B cell responses against specific HLA antigens. This is a two-part, multi-centre, prospective clinical trial, comprising an observational phase (Part 1) aiming to identify patients with unregulated cellular memory responses to HLA (Pure HLA Proteins) followed by an interventional phase (Part 2). The first 9 patients identified as being eligible in Part 1 will undergo baseline immune monitoring for 2 months to inform statistical analysis of the primary endpoint. Part 2 is an adaptive, open labelled trial based on Simon’s two-stage design, with 21 patients receiving Good Manufacturing Practice (GMP)-grade polyclonally expanded Tregs to a dose of 5–10 × 106 cells/kg body weight. The primary EP is suppression of in vitro memory responses for 2 months post-infusion. 12 patients will receive treatment in stage 1 of Part 2, and 9 patients will receive treatment in stage 2 of Part 2 if ≥ 50% patients pass the primary EP in stage 1.

**Discussion:**

This is a prospective study aiming to identify patients with unregulated cellular memory responses to Pure HLA Proteins and determine baseline variation in these patterns of response. Part 2 will be an adaptive phase IIa clinical trial with 21 patients receiving a single infusion of GMP-grade polyclonally expanded Tregs in two stages. It remains to be demonstrated that modulating memory alloresponses clinically using Treg therapy is achievable.

**Trial registration:**

EudraCT Number: 2021–001,664-23.

REC Number: 21/SC/0253.

Trial registration number ISRCTN14582152.

**Supplementary Information:**

The online version contains supplementary material available at 10.1186/s12882-023-03157-7.

## Background

Around 30% of patients on the United Kingdom (UK) transplant waiting list are ‘highly sensitised’ (HS) (National Health Service Blood and Transplant (NHSBT) Annual Report Sept 2018), defined by the presence of Human Leucocytes Antigens (HLA) antibodies (Ab) capable of binding > 85% of the donor pool. As a group, these patients suffer two problems. First, they wait longer for an organ offer, as current standard of care is to only offer organs from donors to whom they have no Ab. To address the issue of waiting longer, the UK donor allocation scheme changed in late 2019 to give priority to HS patients. Disrupted by the COVID-19 pandemic, the full impact of this change on these patients is still not clear, but experience with similar changes in the USA indicates that a significant proportion, with the highest level of HLA antibodies, will not benefit from this [1]. UK Transplant Registry (UKTR) data (2020–21) suggests that after 5 years of waiting, 55% are transplanted, 11% have died, 10% become too frail for transplant, and 24% wait longer than 5 years. Therefore, this group of patients need urgent solutions. To help with offers, they can enter ‘delisting’ or ‘desensitisation’ programmes, where low levels of Ab are ignored (‘delisting’), or higher levels of Ab actively removed immediately prior to transplantation ('desensitisation'). Both these approaches widen the pool of organs available for offer and reduce waiting times and we have shown that patients opting for these programmes are not at higher risk of dying compared to waiting for a well-matched organ, which is the only other option for these patients without a living donor [2, 3]. These are risky options with poorer post-transplant outcome and are offered to selected patients by few centres; in the 5 years to March 2018, fewer than 100 cases were done in the UK. In the near future, novel therapies like Imlifidase [4, 5] or neonatal FcReceptors (FcR) antagonists [6] may offer more opportunity to widen the organ pool and reduce waiting times for this group of patients.

The second problem suffered by this group of patients occurs because the strategies to ignore or temporarily remove Ab allow transplantation to proceed, have no impact on memory alloreactive immune responses. Patients undergoing these procedures have higher rates of acute and chronic rejection, and require more immunosuppression, with all the resulting side effects including infection. Importantly, transplants in these patients have a shortened graft half-life [7, 8]. Five-year graft survival for HLA-incompatible transplants in the UK between 1 April 2021 and 31 March 2016 was 85.0%, compared to 93.2% for transplant from directed unrelated donors (NHSBT Annual Report 2020). We know that alloreactive memory T and B cells specific for donor HLA cause these problems in sensitised patients, despite the enhanced induction and maintenance immunosuppression these patients receive.

Regulatory T cells (CD4^+^CD25^+^Foxp3^+^) “Tregs” have unique immunosuppressive properties, essential in modulating immune responses and inflammation. In pre-clinical models, adoptive transfer of autologous Tregs can facilitate indefinite survival of allografts. This has fuelled an interest in their use in clinical transplantation. Our Good Manufacturing Product (GMP) facility has successfully isolated and expanded Tregs from 12 dialysis patients and demonstrated that they can be safely adoptively transferred back after transplantation (‘ONE study’) and Tregs expanded with the same protocol were used in 9 liver transplant patients ('ThRIL') [8–11]. To date, the focus of this approach has been to promote tolerance post-transplantation, but the aim of this study is to use autologous Tregs to suppress anti-HLA memory responses in sensitised patients awaiting transplantation. The rationale of this approached is based on both pre-clinical data [12] and observations from a study involving 43 patients with biopsy-proven chronic antibody mediated rejection (CAMR) [13, 14] where in donor-specific indirect alloreactive IFNgamma (IFNγ) ELISPOTs, ¾ of samples showed evidence of reactivity against donor antigens. In some patients, the presence of CD25^hi^ Tregs was associated with functional regulation of anti-donor responses by B cells, which reverted to a conventional antigen-presentation role when the Tregs were depleted. These findings have been validated more recently in the 62 patients recruited to the RituxICAN-C4 trial [15]. Thus, Tregs in some patients with CAMR appeared to be modulating anti-donor reactivity, potentially via an effect on antigen-specific B cells and/or T cells. Recently, we associated these patterns of in vitro regulation by CD25^+^ cells, with improved prognosis after 3-year follow-up [14].

In preliminary analyses, we have documented similar patterns in sensitised dialysis patients who have rejected a previous transplant, suggesting that even after the cessation of immunosuppression, patients maintain the ability to regulate responses to specific donor antigens, even though they have made an Ab to that antigen. All these data suggest that augmentation of Treg numbers in patients with HLA Ab may potentially inhibit T &B cell memory responses via interactions with either T or B cells. In support of this, Tregs infused into liver transplant patients in the ThRIL study (at doses lower than we are planning here), have been shown to suppress in vitro ‘direct’ anti-donor responses [9]. Therefore, our hypothesis is that augmentation of Treg numbers in sensitised patients will suppress T cell anti-HLA memory responses for 2 months at least, a period of time that we assess will be clinically useful. The stability or variability of anti-HLA responses over time in patients with end stage renal failure (ESRF) remains unknown, as no-one has studied serial antigen-specific responses in these patients. Important questions relate to whether suppression by Tregs varies spontaneously, and whether it is possible to identify differences induced by Tregs from this background variation.

In this trial, after having identified 21 patients with reactivity against soluble HLA (sHLA) from Pure Protein® (Part 1), we will conduct an adaptive Phase IIa clinical trial (Part 2). The first 9 patients identified as being eligible for Part 2 of the study will undergo baseline immune monitoring for 2 months to inform statistical analysis of primary endpoint. In Part 2 of the study, 12 patients will receive a single infusion of GMP grade polyclonally expanded Tregs in stage 1. After assessing the responses of these for futility/efficacy, we will then administer Tregs to the remaining 9 patients in stage 2 if ≥ 6 patients pass the primary EP in stage 1. The primary EP is suppression of in vitro memory responses for 2 months post-infusion.

## Methods/ design

### Study design

**Fig. 1:**
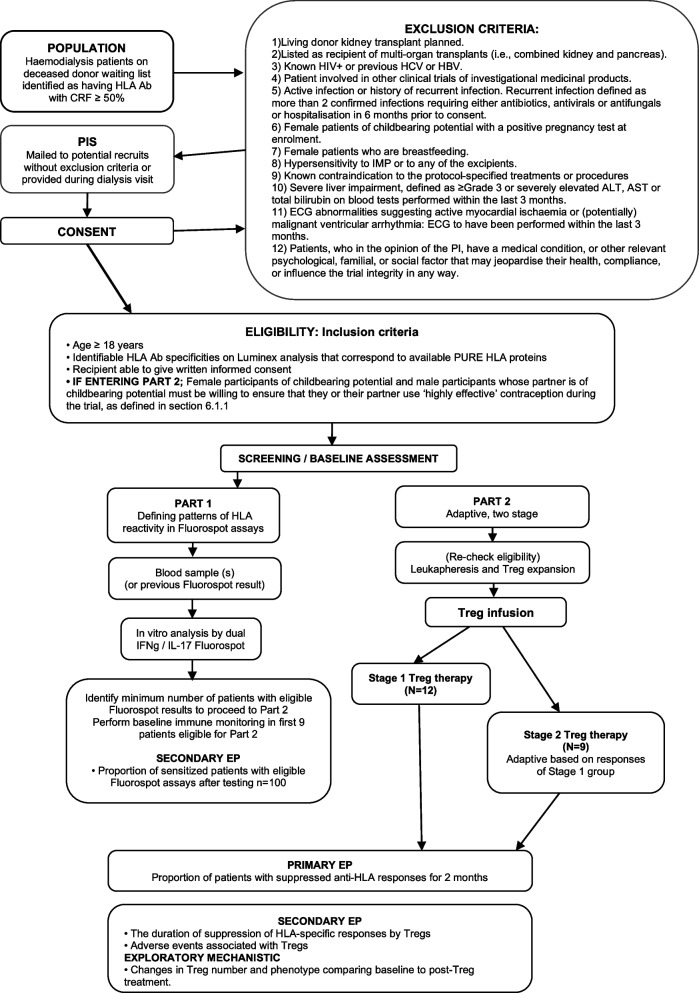
Trial Flow chart Part 1 Observational study: Identification of patients eligible for Part 2, based on pattern of IFNg/IL-17 reactivity on FluoroSpot assay. Some patients may have already had these assays performed in 2020 as part of an observational study (REC ref 16/WM/0370). Additional patients (up to n = 100) will be recruited and have blood withdrawn for testing to determine HLA-specific patterns of responsiveness in FluoroSpot assays, to identify those eligible for Treg therapy in Part 2, and to determine the size of the population for our proposed follow-on trial (beyond this study). All patients with either positive IL-17 or IFN gamma anti-HLA reactivity without evidence of regulation by CD25 + cells will be eligible to enter Part 2. The first 9 patients identified as being eligible, from any site, will undergo baseline immune monitoring for two months to inform statistical analysis of primary endpoint. If < 21 patients with Fluorospot patterns eligible for inclusion in Part 2 are identified, the study will terminate early. Part 2 Interventional Study: This is a two stage, adaptive, open labelled, trial based on Simon’s two stage design, with 12 patients treated in Part 2 Stage 1 and 9 treated in Part 2 Stage 2. Because of an expected high withdrawal rate, all patients recruited to Part 1 will consent to inclusion in Part 2. If more than 21 eligible patients are identified, the statisticians will draw up an algorithm at the end of milestone 1 to determine which patients are included in Part 2, to ensure that eligible patients from each of the three sites are included in a ratio proportionate to the number found eligible from each site. For instance, if after 75 patients have been analysed in Part 1, the number found eligible at each site is 15 (site 1), 10 (site 2) and 10 (site 3), then 9 from site 1 will be included, and 6 each from sites 2 and 3. The order of Treg dosing will be determined by the order in which eligibility for Part 2 was determined. Treg dosing: The exact number in Part 2 Stage 2 who receive Tregs will be determined by the results from Part 2 Stage 1, according to Simon’s 2 stage design. If fewer than 2 of the 12 in stage 1 reach primary endpoint (suppression of anti-HLA responses for at least two months post treatment), the trial will be stopped, concluding that Treg therapy has no efficacy. If 6 or more of the 12 in group 1 reach primary endpoint, we will proceed with dosing all 9 in stage 2. In the event that 3, 4 or 5 of the 12 patients in stage 1 respond, the estimates of efficacy are below our hoped for 50% but are not so low as to be regarded as evidence of futility. We will therefore pause the trial and re-estimate the number of patients that we need to recruit, after discussion with the funders. We anticipate withdrawals from both Part 1 and Part 2, for various reasons including transplantation, and so will likely enrol more than 21 patients to Part2

The trial flow chart is presented in Fig. 1. The study has two parts: Part 1 will be observational to record functional responses by patient peripheral blood mononuclear cells (PBMC) to sHLA from Pure HLA Proteins ® in FluoroSpot assays. The pattern of these responses forms the basis of eligibility to Part 2. The first 9 patients identified as being eligible will undergo baseline immune monitoring for 2 months to inform statistical analysis of primary endpoint. Part 2 is an adaptive, multi-centre, open-label, one-armed, phase IIa clinical trial, based on Simon’s two stage design, with 12 patients treated in stage 1 and 9 treated in stage 2.

### Study participants

Adults with kidney failure awaiting transplantation will be identified from the local kidney transplant waiting list at 3 UK hospitals. The direct care team will approach potential participants and patient information leaflets, describing parts 1 and 2 handed out, emailed or posted to the patient after permission to do so has been obtained. Participants will be offered the opportunity to discuss the trial with the recruiting team. Patients will be asked to provide written consent to inclusion in both Part 1 and Part 2 of the study. Following consent, full eligibility criteria will be reviewed.

### Inclusion criteria

The inclusion criteria for Part 1 are listed as follows:

- 1) Adult (≥ 18yrs) dialysis on the deceased donor renal transplant waiting list with HLA Ab and a Calculated Reaction Frequency (CRF) ≥ 50%
- 2) HLA Ab specificities corresponding to available sHLA from Pure Protein.
- 3) Able to give written informed consent.
- 4) All females of childbearing potential (which is strictly defined in the protocol) and males whose partner is of childbearing potential must be willing to use highly effective methods of contraception if going into Part 2 and continue to use to the end of phase 2 follow-up. Highly effective methods of contraception are strictly defined in the protocol.

Additional inclusion criteria will be confirmed before Part 2 of the study:

- 1) Dual FluoroSpot assay result to sHLA from Pure HLA Proteins® that indicates anti-donor reactivity without evidence of suppression by CD25^+^ T cells in Part 1 (spot count increase, after depletion of CD25 + cells of < 20%).
- 2) Female participants of childbearing potential and male participants whose partner is of childbearing potential must be willing to reconfirm that they or their partner use highly effective contraception during Part 2 of the trial.

### Exclusion criteria

The exclusion criteria for Part 1 are defined as follows:

- 1) Living donor kidney transplant planned.
- 2) Listed as recipient of multi-organ transplants (i.e. combined kidney and pancreas).
- 3) Known Human Immunodeficiency virus (HIV) + or previous Hepatitis C Virus (HCV) or Hepatitis B Virus (HBV). If no HIV, HCV or HBV tests within 5 years, these will be performed post-consent.
- 4) Patient involved in other clinical trials of investigational medicinal products (IMPs).
- 5) Active infection or history of recurrent infection. Recurrent infection defined as more than 2 confirmed infections requiring either antibiotics, antivirals, antifungals or hospitalisation in 6 months prior to consent.
- 6) Female patients of childbearing potential with a positive pregnancy test at enrolment.
- 7) Female patients who are breastfeeding.
- 8) Hypersensitivity to IMP or to any of the excipients.
- 9) Known contraindication to the protocol-specified treatments or procedures.
- 10) Severe liver impairment, defined as ≥ Grade 3 or severely elevated ALT, AST or total bilirubin, on bloods done within the last 3 months.
- 11) Electrocardiogram (ECG) abnormalities suggesting active myocardial ischaemia or (potentially) malignant ventricular arrhythmia: ECG to have been performed within the last 3 months.
- 12) Patients, who in the opinion of the PI, have a medical condition, or other relevant psychological, familial or social factor that may jeopardise their health, compliance, or influence the trial integrity in any way.

Exclusion criteria 1 and 4–12 will be re-checked at a screening visit prior to patient entering Part 2.

### Trial objectives

#### Primary objective

The primary objective is to investigate whether in vitro anti-HLA T & B cell responses in sensitised patients can be inhibited by adoptive transfer of Tregs.

#### Secondary objectives:

1. Determine the proportion of sensitised dialysis patients who may be eligible for a future trial based on patterns of IFNγ/IL-17A responses to HLA on FluoroSpot, i.e. the proportion of patients with unregulated T & B cell anti-HLA responses
2. Determine the duration of suppression of HLA-specific FluoroSpots by Tregs.
3. Determine what adverse events associate with Treg therapy.

The secondary exploratory mechanistic objectives are:

4- Determine how adoptive Treg therapy changes the number and phenotype of circulating Tregs comparing baseline to post-Treg treatment.
5- Determine how adoptive Treg therapy changes HLA Ab profiles measured by Luminex.

##### Patient and public involvement

Members of the local Kidney Patients Association reviewed parts of the grant application to the Medical Research Council and provided feedback on how it could be improved. They were particularly helpful in ensuring the lay summary was written in a helpful way. A patient representative has been invited into the Trial Steering Committee. The findings of the study will be publicised in the Kidney Patients Association Newsletter, website and Facebook.

### Expected duration of trial

The end of the trial is defined as database lock (following completion of monitoring of the last patient last visit undergoing the GAMECHANgER-1 trial).

### Trial endpoints

Primary endpoints.

The primary endpoint is to determine the proportion of patients showing suppression of a defined HLA-specific IFNγ/IL-17A dual FluoroSpot response for 2 months post-treatment, compared to the proportion of patients who show the same changes during baseline immune monitoring. The production of only one cytokine (the one positive at enrolment) will need to be suppressed.

Secondary endpoints.

The secondary endpoints are:

1. Proportion of sensitised dialysis patients with unregulated T & B cell anti-HLA responses, identified in Part 1 observations.
2. Duration of suppression of HLA-specific responses by Tregs.
3. Adverse events associated with Treg therapy.

The secondary exploratory mechanistic endpoints are:

4. Changes in Treg number and phenotype comparing baseline to post-Treg treatment, studied using detailed immunophenotyping performed by flow (and in selected samples where possible mass cytometry) using validated panels and protocols. Correlations between Treg numbers / phenotype and immune reactivity will be sought.
5. The changes in HLA Ab profiles measured by Luminex beads will be assessed using samples taken at enrolment to the observational phase in all, then week 1, 4, 8 then 6- & 12-months post-treatment in those receiving Tregs and baseline, week 1, 4, 8 in those undergoing baseline immune monitoring. All time points ± 1 dialysis session with blood taken at the beginning of dialysis.

### Trial procedures (see Table 1)

**Table 1:** Study Flow Sheet—Summary of trial procedures

	**Part 1 (Visit 1&2 ** ***n*** ** = 100: Visit 3–7 ** ***n*** ** = 9)**								**Part 2 (Stage 1 ** ***n*** ** = 12; Stage 2 ** ***n*** ** = 9)**							
Assessment	Visit 1	Visit 2	Visit 3	Visit 4	Visit 5	Visit 6	Visit 7	Pre-Part 2 eligibility re-check	Visit 1 leukapheresis	Visit 2 Treg infusion	Visit 3	Visit 4	Visit 5	Visit 6	Visit 7	Visit 8
Visit windows Part 1—Relative to Visit 3	See below			+ 1 week ± 1 dialysis session	+ 2 weeks ± 1 dialysis session	+ 4 weeks ± 1 dialysis session	+ 8 weeks ± 2 dialysis sessions	Within 2 weeks of visit 1 Part 2								
Visit windows Part 2—Relative to Visit 2									See below	See below	+ 1 week ± 1 dialysis session	+ 2 weeks ± 1 dialysis session	+ 4 weeks ± 1 dialysis session	+ 8 weeks ± 2 dialysis sessions	+ 6 months ± 3 dialysis sessions	+ 12 months ± 3 dialysis sessions
**Part 1 (** ***n*** ** = 100)**																
Registration Form & Consent	x															
Check / Re-check Inclusion/Exclusion Criteria	x							x								
Post-consent eligibility tests in some recruits*																
HIV, HepBSAg, HepC	x															
Liver function tests	x							x								
ECG	x							x								
Pregnancy test	x							x								
Medical and transplant history, including route of HLA sensitisation	x							x								
Record / update demographic data	x							x								
Blood sample for in vitro dual Fluorospot (in all)	x															
Inform patient of Fluorospot pattern (End of study if ineligible for Part 2)		x														
Baseline Immune monitoring (in first 9 recruits with fluorospot pattern eligible for Part 2) (*n* = 9)			x	x	x	x	x									
Physical exam								x								
Vital signs: pulse, temperature, BP			x	x	x	x	x	x								
Monitoring of AE and IME related to study procedures (i.e. blood test)		x	x	x	x	x	x	x								
**Part 2 (Stage 1 ** ***n*** ** = 12; Stage 2 ** ***n*** ** = 9)**																
Leukapheresis									x							
Symptoms-directed physical exam†									x	x	x	x	x	x	x	x
Vital signs: pulse, temperature, BP									x	x	x	x	x	x	x	x
Blood tests according to EU Tissues and Cells Directive #									x							
Treatment Immune monitoring										x	x	x	x	x	x	x
Full blood count, U&E, LFTs, CRP§										x	x	x	x	x	x	x
Record Concomitant medication										x	x	x	x	x	x	x
Monitoring of AE and IME related to study procedures									x							
Full AE and IME monitoring starts at time of TR001 infusion										x	x	x	x	x	x	x
Pregnancy test																x

Part 1

Visit 1 – At time of recruitment, or next convenient dialysis session if additional post-consent eligibility tests need doing. (Fluorospot only)

Visit 2 – Once visit 1 fluorospot results available No blood tests

Visit 3 – Week ‘0’. Once conditions to proceed with baseline immune monitoring have been met (see Sect. 7.1) (Fluorospot, HLA Ab, Treg numbers and phenotype) –

Visit 4 – 1 week after visit 3 ± 1 dialysis session (Fluorospot, HLA Ab, Treg numbers and phenotype)

Visit 5 – 2 weeks after visit 3 ± 1 dialysis session (Fluorospot only)

Visit 6 – 4 weeks after week 0 ± 1 dialysis session (Fluorospot, HLA Ab, Treg numbers and phenotype)

Visit 7 – 8 weeks after week 0 ± 2 dialysis sessions (Fluorospot, HLA Ab, Treg numbers and phenotype)

Pre-Part 2 eligibility re-check – to be performed approximately 2 weeks prior to leukapheresis

Part 2

Visit 1 – Leukapheresis – at a time convenient for the patient

Visit 2 – Treg infusion – at a time convenient for the patient once Tregs are ready – blood for Fluorospot assay, HLA Ab and determination of Treg numbers and phenotype taken pre-Treg infusion – Sample may be taken at the dialysis session before Treg infusion

Visit 3 – 1 week after Treg infusion ± 1 dialysis session (Fluorospot, HLA Ab, Treg numbers and phenotype)

Visit 4 – 2 weeks after Treg infusion ± 1 dialysis session (Fluorospot)

Visit 5 – 4 weeks after Treg infusion ± 1 dialysis session (Fluorospot, HLA Ab, Treg numbers and phenotype)

Visit 6 – 8 weeks after Treg infusion ± 2 dialysis sessions (Fluorospot, HLA Ab, Treg numbers and phenotype)

Visit 7 – 6 months after Treg infusion ± 3 dialysis sessions (Fluorospot, HLA Ab)

Visit 8 – 12 months after Treg infusion ± 3 dialysis sessions (Fluorospot, HLA Ab)

^*^ Post-consent eligibility tests in some recruits—Take HIV, HepBSAg, Hep C in all unless negative test results are available in last 5 years. Take LFTs in all unless results are available in last 3 months. Do ECG unless a SOC ECG within the last 3 months is available for review. Do pregnancy test on all women of child-bearing potential

^*^ Repeat eligibility tests in some patients at pre-Part 2 assessment. Take LFTs in all unless results are available in last 3 months. Do ECG unless a SOC ECG within the last 3 months is available for review. Do pregnancy test on all women of child-bearing potential

^†^ In all the post-TR001 infusion visits, the need for a symptoms-directed physical examination and/or symptom-specific tests will be determined according to standard medical care

^#^ Mandatory serological tests (Anti-HIV-1, 2, HBsAg, Anti HBc, Anti-HCV-Ab, *T. pallidum-*specific test) are supplemented with HTLV-I/II antibody testing. Window for performing is within 7 days after leukapheresis

^§^ Send sample for FBC, U&E, LFTs and CRP at every visit, unless results available from a dialysis session within the visit window

Visit Schedule and immune monitoring tests

Blood samples will be collected, processed and stored as previously published [13–15]. IFNγ/ IL-17A dual FluoroSpots will be used to describe the patterns of responsiveness to soluble HLA molecules (Pure HLA Proteins®). This assay will be performed on samples from all time points. HLA Ab profiles will be analysed by Luminex. This assay will be performed on samples from all time points except Week 2 of baseline immune monitoring, and Week 2 post-Treg infusion. Flow cytometry (and in selected samples mass cytometry) will be used to analyse phenotypes of circulating Tregs. This will be performed at selected time points. All non-routine laboratory assays will be performed according to Standard Operating Procedures (SOPs).

The Dual Fluoropsot assay will be the FluoroSpot^FLEX^ human IFNγ/ IL-17A dual colour FluoroSpot assay, for simultaneous assessment of both cytokines produced by human CD8-depleted PBMC after incubation with selected sHLA from Pure Protein. Absolute Treg numbers and phenotype will be analysed according to standard flow cytometric protocols in the Core facilities of Guy's Hospital Biomedical Research Centre. Where possible, and dependent on flow cytometry results, selected samples will be tested by mass cytometry in the same facility. The assays to determine HLA Ab profiles will be the LABScreen mixed beads for Class I and II antibody detection, followed by LABScreen single antigen HLA Class I and Class II beads for antibody definition (One Lambda, CA), which will then be analysed on the xMAP Luminex platform. This will be performed at the Viapath Clinical Transplantation Laboratory of Guy’s Hospital using validated protocols.

### Description of the IMP

The Treg product, known as TR001 is classified as an Advanced Therapy IMP (ATIMP). It consists of autologous Tregs isolated from the peripheral blood of recruits by leukapheresis, polyclonally expanded ex vivo, then administered as a single use named patient therapy via intravenous infusion. Cells will be subjected to quality control assessments (immunophenotyping, sterility testing, mycoplasma, endotoxin, and viability) before use. The cell dose is 5–10 × 10^6^ cells/kg.

### Treg acquisition, expansion and administration

- Treg acquisition and expansion

We have developed a GMP-compliant manufacturing process for the production of functionally suppressive TR001 [7]. Autologous PBMC are acquired by leukapheresis. Initial Treg isolation from PBMC consists of depletion of CD8^+^ T cells followed by enrichment for CD25^+^ T cells to obtain a CD4^+^CD25^+^ T cell population, which is subsequently expanded to provide sufficient cell yield. Tregs will be polyclonally expanded based on previous published and validated protocols [8–11, 16–20] in the presence of anti-CD3/CD28 beads, rapamycin (sirolimus) and the growth factor interleukin-2 (IL-2). The Tregs products will follow the same quality control steps prior to release as for the ONE study [10] and described in [8], ie ≥ 60% of CD4 ^+^CD25^+^FOXP3^+^ cells and > 80% suppressive ability in a polyclonal suppression assay. The post-infusion immunological outcomes in UK patients enrolled in the ONE study have been published [11]. We will administer 5–10 × 10^6^ cells/kg, as this was the highest dose safely administered to similar patients in the ONE study [10] without adverse effects and is the dose that has been authorised for use by the TWO Study [21]. Only autologous Treg cells will be utilized in the study participants enrolled in the current trial.

- Treg administration

The TR001 will remain cryopreserved throughout the delivery steps. The cell product will be thawed at the bedside by appropriately trained staff and delivered by syringe into 50mls 4.5% sterile isotonic human serum albumin contained in a glass bottle or proprietary transfer bag. The final dispersed cell product (100mls) will then be infused intravenously by pump by a consultant or similarly qualified medical practitioner. It is anticipated that the infusion will take approximately 30 min.

### Statistical considerations and analysis

#### Sample Size calculation

Based on our previous experience of numbers of patients presenting anti-donor reactivity without evidence of suppression by CD25^+^ T cells, we have calculated that 100 patients will need to be recruited for Part 1. This number is based on availability of the clinical population in the study sites which is feasible in the study timeframe. For Part 2, Simon’s two-stage design will be used for the trial, with α = 0.05 and 1-β = 90%. Clinical consensus amongst the expert team is that if fewer than 20% meet the primary endpoint there is no merit in further investigating Tregs, whereas if ≥ 50% respond to Tregs further investigation will be merited. Based on these parameters, 21 patients are required, all of whom will be monitored for at least 2 months post-treatment to meet the data collection for the primary endpoint. Of these 21 patients, 12 will be allocated to stage 1 and 9 to stage 2, based on the order of recruitment. At the end of stage 1, data will be assessed for futility using stopping rules based on the clinical consensus: ≥ 6 patients meeting the primary endpoint, stage 2 will commence; ≤ 2 patients meeting the primary endpoint, the trial will stop; 3–5 patients meeting the primary endpoint, the trial will pause for the sample size to be re-estimated after discussion with the funder.

#### Primary and secondary analysis

Analysis will be primarily descriptive and focuses upon estimation with 95% confidence intervals. Continuous variables will be summarised using means and standard deviations (if normally distributed, assessed by inspection of histograms) or medians and interquartile ranges. Categorical data will be reported as numbers and frequencies. We will report the proportion of eligible patients tested in Part 1 and the baseline characteristics including route of sensitisation for patients deemed ineligible for Part 2, eligible and treated, and eligible but not treated in Part 2.

We will compare the overall response rate of all 21 Treg-treated patients with control responses rates from the 9 patients undergoing immune monitoring in Part 1. Mixed effects regression models will be utilised to describe and analyse outcomes measured repeatedly over time, with log or other transformation made where necessary to meet model assumptions.

Whilst every endeavour will be made to minimise missing data it is inevitable that there will be some. We will report this by study group and report reasons for missingness where available. We will report data in accordance with the Intent to Treat Principal where all treated patients are included in the relevant parts of the data analysis but also intend to report subgroup analyses in those patients who complete TR001 administration and those who received only a partial dose of TR001 due to medical reasons (if applicable).

Further detail will be reported in the statistical analysis plan.

### Data Handling and management

Participant data will be pseudo-anonymised and will be stored on a password protected computer at each NHS recruiting site. The main dataset is the Elsevier Macro 4 EDC system dataset hosted by the King’s College Clinical Trial Unit. Baseline and follow up data will be collected onto source data worksheets, which will form part of the NHS medical notes. Clinical and research data will be transcribed from the medical notes and source data worksheets to the study eCRF system.

Data relating to the exploratory mechanistic endpoints will be treated differently. A copy of the raw unmanipulated data, labelled with data, PIN and type of analysis will be stored securely as soon as possible after obtained. Descriptive analyses will be prepared from cleaned data by the trial statisticians. Outlying data will be compared to raw data files for validation.

All trial data will be stored in line with the Medicines for Human Use (Clinical Trials) Amended Regulations 2006 and the UK General Data Protection Regulation (UK-GDPR) and UK Data Protection Act 2018 and archived in line with the Medicines for Human Use (Clinical Trials) Amended Regulations 2006 as defined in the Clinical Trials Office Archiving SOP.

### Data monitoring

Risk-based monitoring of this study to ensure compliance with Good Clinical Practice and scientific integrity will be managed by KHP CTO (King’s Health Partners Clinical Trials Office). A Data monitoring Committee including independant related members has been designated to oversee the monitoring of the study. A Trial Steering committee has been designated to provide overall supervision for the GAMECHANgER-1 trial on behalf of the Trial Sponsor and the Trial Funder and to ensure that the trial is conducted according to the guidelines for Good Clinical Practice (GCP), Research Governance Framework for Health and Social Care and all relevant regulations and local policies.

### Dissemination

Results from this study will be published in international peer-reviewed journal with high impact. They will be presented in national, international conferences and using social media. Recruiting sites will be informed of the results and will be asked to disseminate the findings to participants. Patient groups will be informed of the results for dissemination among their members.

## Discussion

The current dogma is that memory immune alloresponses cannot be modulated or suppressed by regulatory T cell [22, 23]. Data from our group [10–12] and others [9] have demonstrated that regulatory T cells could be able to modulate response to HLA in pre-sensitised individuals with CAMR, and that more importantly they are able to fine-tune the phenotype of B cells, allowing them to switch from APC to B cells with regulatory impact. Some recent breakthroughs, including Imlifidase [20] and FcRn antagonists [4], could change the outcomes of antibody incompatible transplantation in the future by targeting humoral responses, however there is no efficient therapy targeting memory immune alloresponses in these patients.

Infusion of polyclonally expanded Tregs has been demonstrated to be safe [8–11], however the ultimate aim in these initial studies was to induce tolerance. Our hypothesis is based on the idea that Treg infusions could modulate memory immune alloresponses. We propose to infuse GMP-grade in vitro expanded polyclonal Tregs into appropriate sensitised patients on the transplant waiting list. Appropriate in this context means those identified in an initial observational phase (Part 1) to have CD4^+^ T cell IFNγ or IL-17A FluoroSpot responses to a defined HLA without evidence of regulation by endogenous Tregs. The HLA antigens used will be those to which the recipient has Ab; to be clinically relevant, we will focus on Ab that could be delisted or be the focus of desensitisation. We anticipate that the Tregs will induce a reduction in the measurable response to the specific anti-HLA B cell-dependent T cell responses, which we will measure using purified HLA antigens in CD4^+^ T cell FluoroSpot assays.

Assuming we demonstrate that our strategy suppresses responses in significant proportions of patients, an important question is how long does the effect last? This will be evaluated using data from immune monitoring post-Treg infusion. We will use this information to model, with NHSBT, the impact of the new allocation scheme on numbers of highly sensitised patients, in conjunction with early data on their observed post-transplant outcomes. In addition, with the advent of novel therapies like Imlifidase [20] and FcRn antagonists [4], we will model the impact of delisting or desensitising significant numbers of new patients, to inform on the feasibility of performing a larger trial, with clinically relevant endpoints in these patients.

Our ultimate goal is to develop non-toxic immunotherapies capable of controlling cell-mediated B and T cell responses in these ‘highly sensitised’ patients to promote better graft and patient outcomes and ultimately better access to transplantation in this group. This trial will deliver the insights necessary to assess whether further clinical investigation / application of our solution is feasible.

### Trial status

This manuscript is based on protocol version2, 6/12/21. At the time of submission, the trial has recruited 31 patients to Part 1, after recruitment started in April 2022.

## Supplementary Information

**Additional file 1.**

## Data Availability

Not applicable.

## Abbreviations

ATIMP: Advanced Therapy IMP
Ab: Antibodies
CRF: Calculated Reaction Frequency
CAMR: Chronic antibody mediated rejection
ESRF: End stage renal failure
FcR: FcReceptors
GMP: Good Manufacturing Practice
HS: Highly sensitised
HCV: Hepatitis C Virus
HBV: Hepatitis B Virus
HIV: Human Immunodeficiency virus
HLA: Human leukocyte antigens
KHP CTO: King’s Health Partners Clinical Trials Office
IFNγ: IFNgamma
IMPs: Investigational medicinal products
NHSBT: National Health Service Blood and Transplant
NIHR: National Institute for Health Research
PBMC: Peripheral blood mononuclear cells
Tregs: Regulatory T cells
Shla: Soluble HLA
UKTR: UK Transplant Registry

## References

[1] Schinstock CA, Smith BH, Montgomery RA, Jordan SC, Bentall AJ, Mai M (2019). Managing highly sensitized renal transplant candidates in the era of kidney paired donation and the new kidney allocation system: Is there still a role for desensitization?. Clin Transplant.

[2] Manook M, Koeser L, Ahmed Z, Robb M, Johnson R, Shaw O (2017). Post-listing survival for highly sensitised patients on the UK kidney transplant waiting list: a matched cohort analysis. The Lancet.

[3] Couzi L, Manook M, Perera R, Shaw O, Ahmed Z, Kessaris N (2015). Difference in outcomes after antibody-mediated rejection between abo-incompatible and positive cross-match transplantations. Transpl Int.

[4] Jordan SC, Lorant T, Choi J, Kjellman C, Winstedt L, Bengtsson M (2017). IgG Endopeptidase in Highly Sensitized Patients Undergoing Transplantation. N Engl J Med.

[5] Kjellman C, Maldonado AQ, Sjöholm K, Lonze BE, Montgomery RA, Runström A (2021). Outcomes at 3 years posttransplant in imlifidase-desensitized kidney transplant patients. Am J Transplant.

[6] Patel DD, Bussel JB (2020). Neonatal Fc receptor in human immunity: Function and role in therapeutic intervention. J Allergy Clin Immunol.

[7] Pankhurst L, Hudson A, Mumford L, Willicombe M, Galliford J, Shaw O (2017). The UK National Registry of ABO and HLA Antibody Incompatible Renal Transplantation: Pretransplant Factors Associated With Outcome in 879 Transplants. Transplant Direct.

[8] Fraser H, Safinia N, Grageda N, Thirkell S, Lowe K, Fry LJ (2018). A Rapamycin-Based GMP-Compatible Process for the Isolation and Expansion of Regulatory T Cells for Clinical Trials. Molecular Therapy Methods & Clinical Development.

[9] Sánchez-Fueyo A, Whitehouse G, Grageda N, Cramp ME, Lim TY, Romano M (2020). Applicability, safety, and biological activity of regulatory T cell therapy in liver transplantation. Am J Transplant.

[10] Sawitzki B, Harden PN, Reinke P, Moreau A, Hutchinson JA, Game DS, et al. Regulatory cell therapy in kidney transplantation (The ONE Study): a harmonised design and analysis of seven non-randomised, single-arm, phase 1/2A trials. Lancet. 2020 23;395(10237):1627–39. 10.1016/S0140-6736(20)30167-7 PMC7613154 32446407

[11] Harden PN, Game DS, Sawitzki B, Van der Net JB, Hester J, Bushell A (2021). Feasibility, long-term safety, and immune monitoring of regulatory T cell therapy in living donor kidney transplant recipients. Am J Transplant.

[12] Carvalho-Gaspar M, Jones ND, Luo S, Martin L, Brook MO, Wood KJ (2008). Location and Time-Dependent Control of Rejection by Regulatory T Cells Culminates in a Failure to Generate Memory T Cells. J Immunol.

[13] Shiu KY, McLaughlin L, Rebollo-Mesa I, Zhao J, Semik V, Terence Cook H (2015). B-lymphocytes support and regulate indirect T-cell alloreactivity in individual patients with chronic antibody-mediated rejection. Kidney Int.

[14] Shiu KY, Stringer D, McLaughlin L, Shaw O, Brookes P, Burton H (2020). Effect of Optimized Immunosuppression (Including Rituximab) on Anti-Donor Alloresponses in Patients With Chronically Rejecting Renal Allografts. Front Immunol.

[15] Shiu KY, McLaughlin L, Rebollo-Mesa I, Zhao J, Burton H, Douthwaite H (2017). Graft dysfunction in chronic antibody-mediated rejection correlates with B-cell–dependent indirect antidonor alloresponses and autocrine regulation of interferon-γ production by Th1 cells. Kidney Int.

[16] Gao W, Lu Y, El Essawy B, Oukka M, Kuchroo VK, Strom TB (2007). Contrasting effects of cyclosporine and rapamycin in de novo generation of alloantigen-specific regulatory T cells. Am J Transplant.

[17] Tian L, Lu L, Yuan Z, Lamb JR, Tam PKH (2004). Acceleration of apoptosis in CD4+CD8+ thymocytes by rapamycin accompanied by increased CD4+CD25+ T cells in the periphery. Transplantation.

[18] Game DS, Hernandez-Fuentes MP, Lechler RI (2005). Everolimus and Basiliximab Permit Suppression by Human CD4 ^+^ CD25 ^+^ Cells *in vitro*. Am J Transplant.

[19] Basu S, Golovina T, Mikheeva T, June CH, Riley JL (2008). Cutting edge: Foxp3-mediated induction of pim 2 allows human T regulatory cells to preferentially expand in rapamycin. J Immunol.

[20] Zeiser R, Leveson-Gower DB, Zambricki EA, Kambham N, Beilhack A, Loh J (2008). Differential impact of mammalian target of rapamycin inhibition on CD4+CD25+Foxp3+ regulatory T cells compared with conventional CD4+ T cells. Blood.

[21] Brook MO, Hester J, Petchey W, Rombach I, Dutton S, Bottomley MJ, Black J, Abdul Wahab S, Bushell A, Lombardi G, Wood K, Friend P, Harden P, Issa F. Transplantation Without Overimmunosuppression (TWO) study protocol: a phase 2b randomised controlled single-centre trial of regulatory T cell therapy to facilitate immunosuppression reduction in living donor kidney transplant recipients. BMJ Open. 2022 Apr 15;12(4):e061864. doi: 10.1136/bmjopen-2022-061864. 10.1136/bmjopen-2022-061864 PMC9014059 35428650

[22] Yang J, Brook MO, Carvalho-Gaspar M, Zhang J, Ramon HE, Sayegh MH (2007). Allograft rejection mediated by memory T cells is resistant to regulation. Proc Natl Acad Sci U S A.

[23] Afzali B, Mitchell PJ, Scottà C, Canavan J, Edozie FC, Fazekasova H, Lord GM, John S, Barber LD, Hernandez-Fuentes MP, Lechler RI, Lombardi G (2011). Relative resistance of human CD4(+) memory T cells to suppression by CD4(+) CD25(+) regulatory T cells. Am J Transplant.

