# Supplementary material for: Can regulatory T cells improve outcomes of sensitised patients after HLA-Ab incompatible renal transplantation: study protocol for the Phase IIa GAMECHANgER-1 trial
Source: BMC Nephrol. 2023 Apr 28;24:117. doi: 10.1186/s12882-023-03157-7 (PMC10140710; doi:10.1186/s12882-023-03157-7)
Supplement: Supplementary file 1 — Additional file 1. [file 12882_2023_3157_MOESM1_ESM.docx]

**Appendix**

**Details of Trials steering Committee**

The role of the Trial Steering Committee (TSC) is to provide overall supervision for the GAMECHANgER-1 trial on behalf of the Trial Sponsor and the Trial Funder and to ensure that the trial is conducted according to the guidelines for Good Clinical Practice (GCP), Research Governance Framework for Health and Social Care and all relevant **regulations** and local policies.

# Terms of reference

- To monitor and supervise the progress of the trial GAMECHANgER-1 towards its interim and overall objectives, review accrual and results of the trial, adherence to the protocol, patient safety and the consideration of new information of relevance to the trial and the research question.
- To review at regular intervals relevant information from other sources, (eg. other related trials).
- To consider the recommendations of the Data Monitoring Committee.
- To report to the Sponsor on progress of the trial, and if necessary to MRC as funder.
- To advise the Principal Investigator, Sponsor and MRC as funder on publicity and the presentation of all aspects of the trial.

# Membership and Primary responsibilities of the TSC

The GAMECHANgER-1 TSC is a multidisciplinary group comprising of the following members who jointly have responsibility for the design, conduct and evaluation of the clinical research project.

- Independent Chair: Dr Sian Griffin
- Chief Investigator Professor Anthony Dorling
- Members of the TMG: Dr Izabela Pilecka (Trial Manager) and one or two from the following co-investigators: Dr David Game, Professor Nizam Mamode, Professor Giovanna Lombardi, Professor Abberto Sanchez-Fueyo
- Independent clinician(s) or Scientist(s) with relevant experience: Dr Brendan Clark
- Representative of relevant patient group: Mr Matthew Sellen

**3. Interaction between TSC and other study committees**

- The TSC will receive reports and requests from the TMG and reports and recommendations from the DMC, particularly pertaining to patient safety
- The TSC will make recommendations to the TMG, including suggestions about communicating with the sponsor and funder, when thought necessary
- The TSC may, in exceptional circumstances, communicate directly with the sponsor or funder

# Responsibilities

The TSC on behalf of the Sponsor and Funder will have overall responsibility for the design and conduct of the trial and for safeguarding the rights, safety and well being of participants. Responsibilities of the TSC/SSC to include:

- Reviewing recruitment/retention of participants and their management
- Reviewing study protocol and other study documentation.
- Determine if amendments to the protocol or changes to study conduct are required and deciding on changes to these and to study conduct in general. Any changes to trial documentation or conduct must be notified to the TSC/SSC.
- Reviewing adherence to the protocol by Investigators and participants
- Assessing the impact and relevance of external evidence
- Assessing integrity and completeness of data collected
- Monitoring the overall conduct of the trial, ensuring that it follows the standards set out in the guidelines of GCP, assessing the safety and efficacy of the interventions, recruitment figures and completion of trial assessments.
- Reviewing, commenting and making decisions on extension requests.
- Reviewing the recommendations of the DMC and suggesting appropriate action to the TMG
- Monitoring the progress of study/trial and deciding on appropriate action in order to maximise the chances of completing it within the agreed timelines.
- Considering new information relevant to the study e.g. results from other studies that may have a bearing to the conduct of the study and deciding on appropriate action.
- Endorsing the annual report to the funder

The TSC may recommend early termination of the trial or modification of the study design in the event of a clear outcome derived from accumulating data or on the basis of information available from other sources or on safety grounds.

The TSC should be available to provide independent advice as required not just when meetings are scheduled.

The TSC should maintain confidentiality of all information it receives.

Members should not discuss confidential issues from their involvement in the study until the primary results have been published.

# Role of the TSC Chair

- Arrange the first meeting of the TSC with the assistance of the trial manager and CI to agree contents of Terms of Reference and set up schedule of meetings
- Establish clear reporting lines – to the Funder, Sponsor etc
- Become familiar with the role of the DMC
- Provide an independent, experienced opinion if conflicts arise between the needs of the research team, the Funder, the Sponsor and/or any other agencies
- Leading the TSC to provide regular, impartial oversight of the trial, especially to identify and pre-empt problems
- Ensuring that changes to the protocol are debated and endorsed by other members of the TSC

For decisions to be made, at least 2 independent members of the TSC should be present (including the chair), the CI and a representative from the TMG.

# TSC meetings

- The responsibility for calling and organising a TSC meeting lies with the CI, through the trial manager, in association with the TSC Chair.
- The trial manager will organise meetings on behalf of the CI
- The meetings will preferably be in person, but circumstances might dictate that teleconferencing is more appropriate/acceptable to committee members
- All TSC members will be provided with study documents (e.g. protocol, proposed statistical analysis plan (SAP), PIS, CRF etc) and the TSC report prior to the meeting.
- The first TSC meeting should ideally be held face-to-face to discuss, revise and finalise the terms of reference, agree the content of the TSC/SSC Terms of Reference and sign any declaration, and agree the frequency of the meetings.
- The frequency of subsequent meetings will be at least six monthly, with additional meeting on meeting milestones in Part 1, and after Stage 1 of Part 2 plus, if required, following a DMC meeting.
- Meetings can also be held at any time at the request of the CI or TSC chair
- The final TSC meeting will be arranged when target recruitment is completed, all data collected and cleaned, and the database is locked. This final meeting will be held to discuss final/completed data and interpretation, and publication timelines. If the study is terminated prematurely, no final study meeting is required.

# Attendance

Every effort will be made to ensure that all TSC members can attend the meetings. The study coordinator or delegate should try and find a date that enables this. The CI must try to attend all meetings, especially if major actions are expected.

The Chair, CI, the Trial Manager and at least one other independent member must be present for a meeting to be regarded as quorate.

If the TSC is considering major actions the TSC Chair should communicate with absent members, including the CI, as soon after the meeting as possible to determine whether they all agree. If there is disagreement amongst absent members a further meeting should be arranged with the full TSC.

# Reporting

Prior to a TSC meeting an open report will be prepared by the trial manager with input from statistician and circulated to TSC members at least a week, as otherwise agreed before the meeting.

On consideration of the information presented at these meetings, the TSC should provide recommendations of appropriate action in writing to the TMG who will be responsible for implementing any actions. The TSC may also provide feedback to the DMC and where appropriate to the Sponsor/Funder.

Minutes of the meeting including key points and actions will be prepared by the Trial Manager. These minutes will describe the proceedings and include the recommendations of the TSC. All members of the TSC must agree the minutes and these will be signed off by the TSC Chair on behalf of all members. Minutes will be circulated to all TSC members, the TMG, the Sponsor and, if appropriate, the Trial Funder. Approved Minutes will be filed in the Trial Master File.

Decisions and recommendations by the TSC should be unanimous if not a vote may be taken. The role of the Chair is to summarise discussions and encourage consensus. Therefore, it is best for the chair to give his own opinion last. It is important that the implications (ethical, statistical, practical, and financial) for the trial be considered before any decision is made.

# Contents of the TSC/SSC Reports

An outline of the contents of the TSC report is given below:

- Outline of the study design, sample size sought and current available evidence
- Statistical consideration and design
- Major protocol amendments
- Patient screening
- Eligibility violations
- Protocol violations by investigators or participants
- Study accrual by month/total
- Completeness and quality of data collected/CRF return
- Quality controls
- CRF return, entry into database
- Baseline characteristics
  - Demographics
  - Disease characteristics
  - Previous treatment usage
  - Laboratory
- Safety reporting
- Follow up data available
- Any matters affecting the trial
- Compliance by patients to clinic visit
- Latest DMC report and DMC recommendations

# Conflicts of interest

TSC members should not have any apparent financial, scientific or intellectual conflict of interest that could prevent them from objectively reviewing the study protocol, interim and final data and giving advice to the TMG. TSC members should disclose to the Chair any other conflicts they consider relevant. Any members who develop significant conflicts of interest during the course of the trial should resign from the TSC.

# Publication

Manuscripts that arise from the trial will be shared with the TSC and members will be able to comment. The TSC members and their affiliations will be acknowledged in reports of the trial.

**Details of the Data Monitoring Committee**

The role of the DMC is to provide overall supervision for the GAMECHANgER-1 trial on behalf of the Trial Sponsor and the Trial Funder and to ensure that the trial is conducted in compliance with the

principles of the Declaration of Helsinki (1996), the principles of GCP and in accordance with all applicable regulatory requirements including but not limited to the Research Governance Framework and the Medicines for Human Use (Clinical Trial) Regulations 2004, as amended in 2006 and any subsequent amendments.

The DMC should receive and review information on the progress and accruing data of this trial and provide advice on the conduct of the trial

**Specific roles of DMC**

Review of the trial’s progress including updated figures on recruitment, data quality, adherence to protocol treatment and follow-up, and main outcomes and safety data. Specifically, these roles include to:

• monitor evidence for treatment harm (e.g. toxicity, SAEs and SARs, deaths)

• assess the impact and relevance of external evidence if any.

• at the end of stage 1 in part 2, decide whether to recommend that the trial continues to recruit participants or whether recruitment should be terminated for futility reasons.

• assess data quality, including completeness (and by so doing encourage collection of high quality data)

• maintain confidentiality of all trial information that is not in the public domain

- monitor recruitment figures and losses to follow-up

• monitor compliance with the protocol by participants and investigators
